# Adsorption and electronic properties of pentacene on thin dielectric decoupling layers

**DOI:** 10.3762/bjnano.8.140

**Published:** 2017-07-06

**Authors:** Sebastian Koslowski, Daniel Rosenblatt, Alexander Kabakchiev, Klaus Kuhnke, Klaus Kern, Uta Schlickum

**Affiliations:** 1 Max-Planck-Institut for Solid State Research, Heisenbergstrasse 1, D-70569 Stuttgart, Germany; 2 Institut de Physique, Ecole Polytechnique Fédérale de Lausanne (EPFL), CH-1015 Lausanne, Switzerland

**Keywords:** hexagonal boron nitride (h-BN), metal insulating organic interface, pentacene, potassium chloride (KCl), scanning tunnelling microscopy (STM), scanning tunnelling spectroscopy (STS)

## Abstract

With the increasing use of thin dielectric decoupling layers to study the electronic properties of organic molecules on metal surfaces, comparative studies are needed in order to generalize findings and formulate practical rules. In this paper we study the adsorption and electronic properties of pentacene deposited onto h-BN/Rh(111) and compare them with those of pentacene deposited onto KCl on various metal surfaces. When deposited onto KCl, the HOMO and LUMO energies of the pentacene molecules scale with the work functions of the combined KCl/metal surface. The magnitude of the variation between the respective KCl/metal systems indicates the degree of interaction of the frontier orbitals with the underlying metal. The results confirm that the so-called IDIS model developed by Willenbockel et al. applies not only to molecular layers on bare metal surfaces, but also to individual molecules on thin electronically decoupling layers. Depositing pentacene onto h-BN/Rh(111) results in significantly different adsorption characteristics, due to the topographic corrugation of the surface as well as the lateral electric fields it presents. These properties are reflected in the divergence from the aforementioned trend for the orbital energies of pentacene deposited onto h-BN/Rh(111), as well as in the different adsorption geometry. Thus, the highly desirable capacity of h-BN to trap molecules comes at the price of enhanced metal–molecule interaction, which decreases the HOMO–LUMO gap of the molecules. In spite of the enhanced interaction, the molecular orbitals are evident in scanning tunnelling spectroscopy (STS) and their shapes can be resolved by spectroscopic mapping.

## Introduction

Miniaturization plays a paramount role in the development of modern technology. In order to further reduce the dimensions of the basic processing units, molecular electronics is a promising approach. To understand the principles behind single-molecule devices, the fundamental physics of molecule-metal junctions need to be well understood and controlled.

Scanning tunneling microscopy (STM) is particularly suited to not only study the structure of an adsorbed (organic) molecule on the atomic scale [1–2] but furthermore to probe its electronic properties [3]. Aromatic molecules deposited onto a metal surface are known to interact through their π-orbitals via van der Waals interactions with the free electron gas at the metal surface [4]. This can lead to a charge-transfer process with or without [5] a chemical reaction between the metal surface and the adsorbed molecule and thus to a hybridization of the molecule with the underlying electron bath of the metal surface. To access the intrinsic electronic structure of molecules using STM, it has been shown that it is in most cases mandatory to sufficiently separate (electronically decouple) the molecules from the underlying metal substrate by means of thin insulating layers (decoupling layers) [6]. To achieve this, a thin spacer layer with a large band gap of several electronvolts can be used that acts as a tunnel barrier towards the metal [7]. For example, it has been shown that NaCl with a thickness of merely one to three atomic layers grown on a Cu(111) surface provides sufficient electronic decoupling between pentacene molecules and the metal surface [6]. The influence of the inert NaCl layer on the molecules is very small, as demonstrated by the observation of the unperturbed gas-phase-like frontier orbitals of pentacene on this substrate [3]. A weak interaction is nevertheless suggested by the observed shift of the orbital energies of the admolecule [4,7–8].

In scanning tunneling spectroscopy (STS) experiments on pentacene, two well-defined increases in the local density of states are observed close to the Fermi level [3–4]. Tunneling through these states of pentacene results in a temporary charging of the molecule prior to the dissipation of the charge into the substrate [5]. The peaks observed in STS on pentacene are therefore also referred to as the positive- and negative-ion resonances (PIR and NIR). The NIR corresponds to the highest occupied molecular orbital (HOMO) and the PIR to the lowest unoccupied molecular orbital (LUMO). Various thin insulating layers have so far been used as decoupling layers in STM experiments [9–12]. It is, however, desirable to classify these systems according to their capability to effectively decouple an organic adsorbate from the underlying metal substrate. In this study pentacene (C_22_H_14_) is used as a model system. We investigate the structural and electronic properties of pentacene on a monoatomic layer of hexagonal boron nitride (h-BN) and compare it to its properties on KCl layers grown on various low-index noble-metal surfaces.

## Results and Discussion

**Figure 1:**
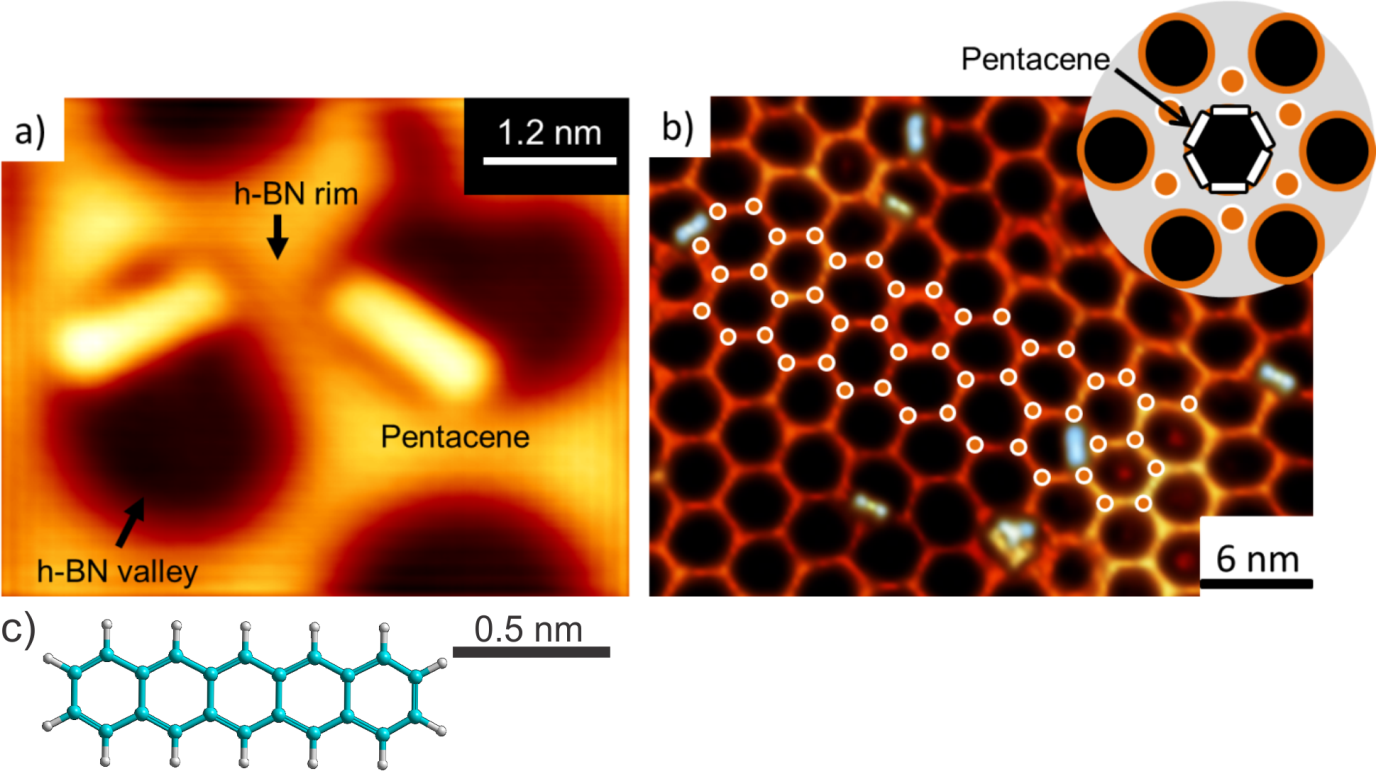
a) STM topography image of pentacene on h-BN (*U* = −1 *V*, *I* = 50 pA). b) Large-scale STM topography image of pentacene on h-BN. The white circles indicate the preferential adsorption sites illustrated in the inset. Inset: Illustration of the six possible pentacene adsorption geometries inside the h-BN valley (*U* = −1 V, *I* = 50 pA). c) Representation of the molecular geometry of a free pentacene molecule.

Here we start with a detailed discussion of the properties of pentacene deposited onto h-BN grown on Rh(111). Due to the lattice mismatch to Rh(111), the h-BN forms a buckled moiré superstructure [12]. The subsequently deposited pentacene molecules are preferentially found at the edges of the valleys of the h-BN moiré superstructure (Figure 1), similarly to what was observed for the CuPc/h-BN/Rh(111) system [13], which was explained by dipole rings inside the valleys. This resulting in-plane electric field attracts the adsorbed molecules to the edges of the valleys.

The pentacene molecules appear as 1.5 nm long rod-like objects with an apparent height of about 1 Å when scanned in a bias range of ±1V (in the molecular HOMO–LUMO gap) (Figure 1a). An overall preferential six-fold adsorption geometry can be observed. The pentacene molecules preferentially adsorb with their long axis facing an intersection of rims (orange dots in inset Figure 1b). Molecules parallel to the h-BN rim are not observed. Geometric conditions, e.g., energy minimization due to best alignment with the hexagonal valley, might hinder additional adsorption geometries. Defects or multiple pentacene molecules adsorbed in the same valley, however, break this scheme and cause adsorptions in alternative geometries.

**Figure 2:**
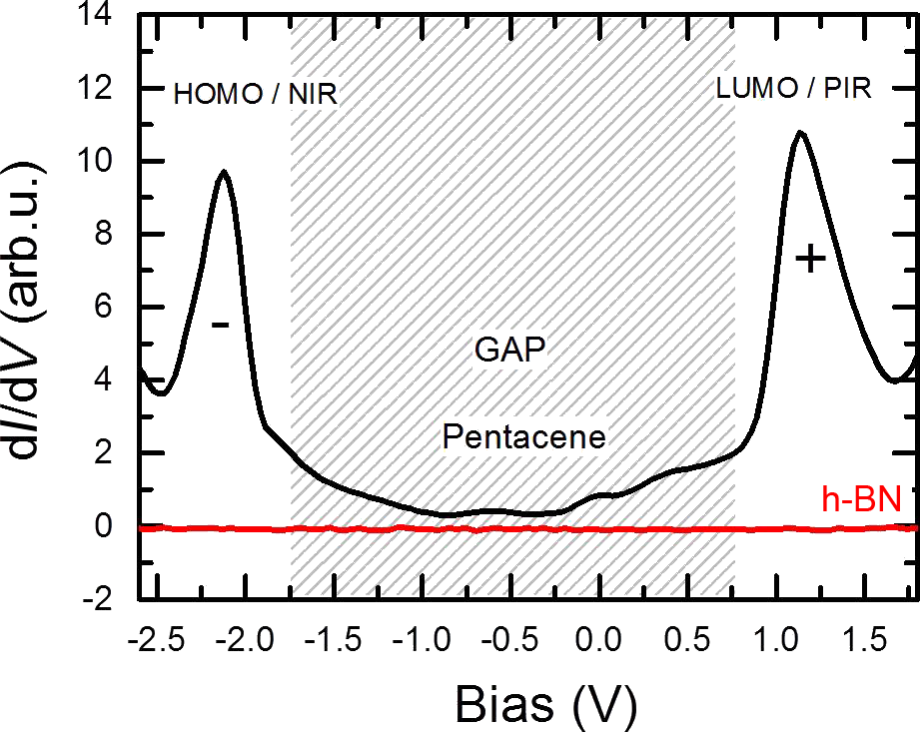
STS of pentacene on h-BN/Rh(111) showing the resonances of the frontier molecular orbitals. The red curve depicts the d*I*/d*V* of bare h-BN. Zero bias refers to the Fermi energy of the Rh substrate.

The electronic properties of the pentacene molecules were probed by measuring the differential conductance (d*I*/d*V*) in STS experiments. In STS, pentacene reveals two molecular orbitals near the Fermi energy of the substrate, one at negative (−2.1 V) and one at positive bias voltage (+1.2 V) (Figure 2). The absolute peak positions showed some variation from molecule to molecule, presumably due to slightly different adsorption geometries. The typical gap between the peaks amounts to 3.39 ± 0.31 V. The positions of the peaks were determined to be −2.16 ± 0.16 V and +1.22 ± 0.22 V. The error was obtained as standard deviation from statistical evaluations of the measured molecules.

**Figure 3:**
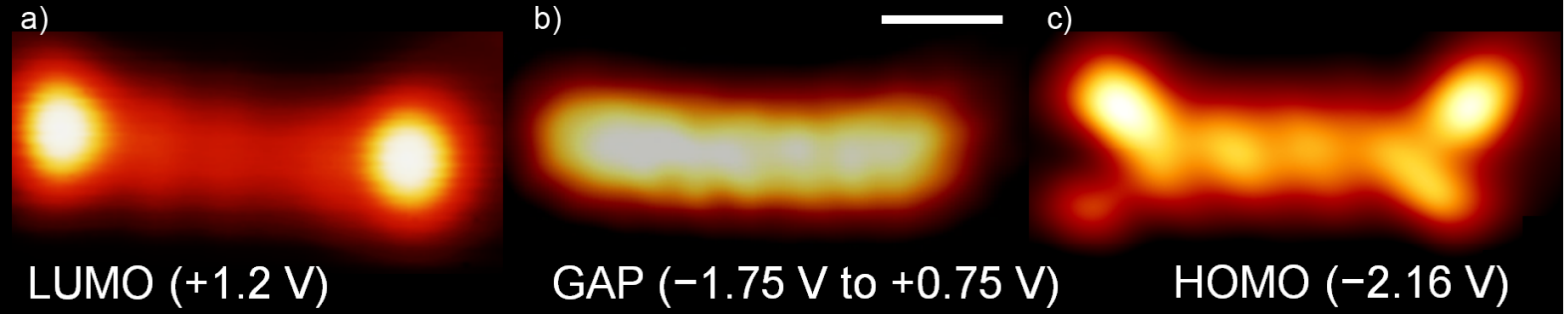
Spatial mapping of the molecular orbitals of pentacene. a) At positive bias voltages (LUMO), b) inside the gap and c) at negative (HOMO) bias voltages (bias voltages are indicated in the image). The scale bar equals 0.5 nm (the voltage is indicated in the images, *I* = 50 pA, constant height).

Spatial imaging at the energies of the orbitals observed in STS yields the spatial distribution of the frontier orbitals of pentacene [2–4]. For +1.2 V, two prominent lobes connected by a series of dimmer lobes are observed (Figure 3a). This structure can be ascribed to the lowest unoccupied molecular orbital (LUMO). For voltages within the range from −1.75 V to +0.75 V, the molecule appears as a featureless rod with low apparent height (Figure 3b). At −2.16 V, we observe the structure of the highest occupied molecular orbital (HOMO) (Figure 3c), with four prominent lobes connected by a row of smaller lobes. The differences in apparent height along the short axis of the molecule can be attributed to the local slope of the adsorption position at the edges of the valley of the h-BN mesh underneath the molecule.

**Figure 4:**
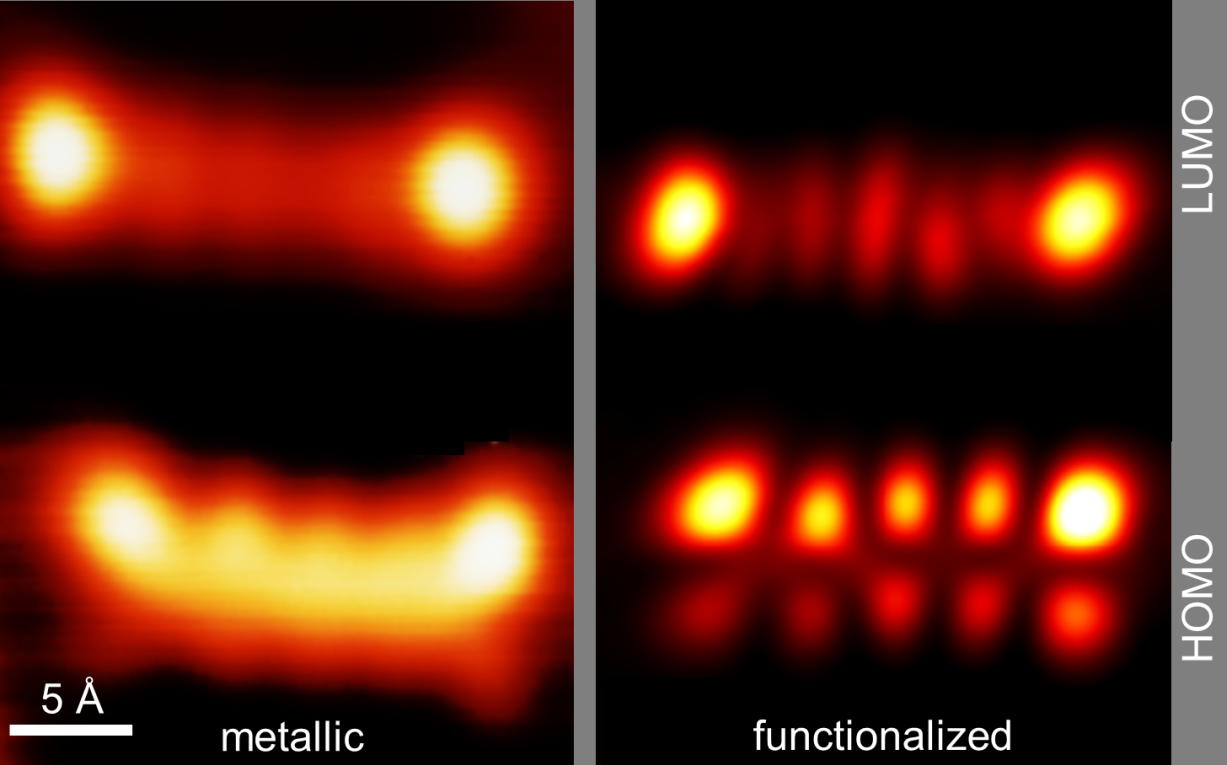
Spatial mapping of the molecular orbitals of pentacene by employing an STM tip without (left) and with (right) pentacene functionalization (*U*(LUMO) = +1.2 V, *U*(HOMO) = −2.1 V, *I* = 50 pA, constant height).

In order to improve the spatial resolution, the tip is functionalized with a pentacene molecule, as has been shown for pentacene on NaCl [3] (Figure 4). The functionalization is achieved by positioning a metallic tip over the endpoint of the pentacene molecule. While monitoring the current, the tip is lowered until an abrupt change in the tunnel current is measured, marking the jump of the molecule to the tip apex. A pentacene-functionalized STM tip might express a considerably smaller effective tip apex due to scanning with one of the π-orbitals of the attached pentacene molecule and thereby improves spatial resolution. Using a functionalized tip, the LUMO is now imaged as a structure with two bright lobes at the ends separated by five smaller elongated lobes with slightly varying shapes. The HOMO exhibits four prominent lobes at the edges and six smaller lobes .The structure has a node along its long axis (Figure 4).

**Figure 5:**
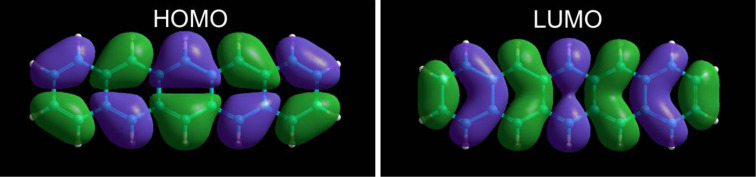
Hückel calculations of the frontier molecular orbitals (HOMO and LUMO) of pentacene in the gas phase superimposed with a ball-and-stick model of the molecular structure.

The assignment of HOMO and LUMO is further supported by Hückel calculations that yield nodal structures similar to the ones observed by STS. The calculated orbital structure consists of a sequence of orbital lobes with alternating sign of the wave function Ψ, indicated by the color code (violet, green) in Figure 5. The experimental observation is in good agreement with the structures obtained from Hückel calculations of the free molecule.

In order to quantify the values observed for the HOMO and LUMO in terms of their energy position, one has to relate them with other measurable quantities of the investigated system. Two well-suited quantities for molecules are the electron affinity (*E*_a_), i.e., the energy cost of adding an electron to the neutral molecule, and the ionization energy (*E*_i_), the energy needed to remove an electron from a neutral molecule [14]. For various molecular species, including pentacene, these values are well known from photoemission and inverse photoemission experiments [15].

**Figure 6:**
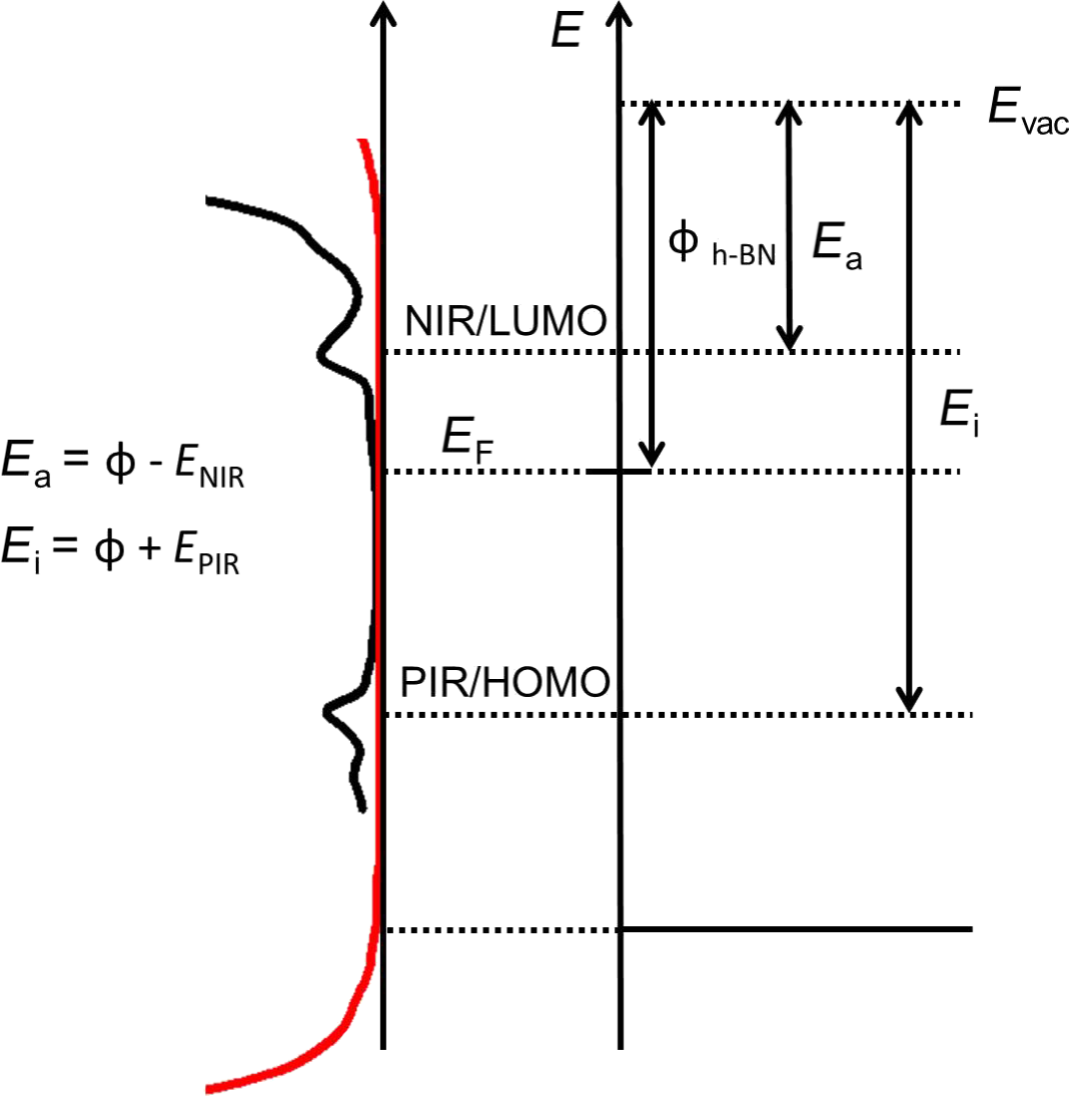
Energy-level diagram depicting the relations between *E*_a_, *E*_i_, Φ and the energies measured in STS for the HOMO and the LUMO of pentacene.

In a first approximation, the physical quantity connecting *E*_a_ and *E*_i_ with the measured energetic positions of the HOMO and LUMO, respectively, is the work function Φ of the underlying h-BN/Rh(111) substrate, namely the energy difference between the vacuum level and the Fermi energy. The specific relation between these quantities is depicted in the scheme in Figure 6.

With Φ = 4.15 eV [16] as a literature value for a single layer of h-BN on Rh(111), the underlying h-BN substrate can be described. With this, the HOMO at −2.16 V can be attributed to a bound state with an energy of 6.31 eV and, respectively, the LUMO at 1.2 V to a bound state with an energy of 2.95 eV. These energies can be related to the electron affinity of 1.35 eV [14] and the ionization energy of gas-phase pentacene at 6.58 eV [15]. The deviation of the gas-phase energies from the energies found on the surface can be explained by the screening of the underlying substrate during the temporary charging of the molecule [1,17–18]. In our measurements we observe a decrease of the HOMO–LUMO gap by 1.7 eV (i.e., an average shift of 0.85 eV for HOMO and LUMO) in comparison to the gas-phase value for pentacene. Thus the molecular ion (positive/negative) is stabilized with respect to the gas-phase ion due to screening of the charge of the molecular ions, induced by the formation of image charges in the topmost layers of the metal substrate, as described by Willenbockel et al. [17]. This is in agreement with photoelectron spectroscopy measurements on pentacene molecules in the gas phase. Measurements on molecules adsorbed on metal surfaces showed that the energies of both the HOMO and the LUMO were shifted towards the Fermi energy, reducing the HOMO–LUMO gap of the molecule.

To estimate the expected reduction of the gap, we propose and discuss a point-charge model. We assume a single charge (*q*_1_ = *q*_2_ = *e*) at a distance 2*d* from its image charge. The distance *d* between the charge (ion) and the metal surface (reflection plane) was estimated to be 2.8 Å using calculations for the h-BN/Rh(111) system based on the local distance of the h-BN layer from the metal substrate at the position of the pentacene molecule [12]. Thereby no electric field caused by an in-plane dipole moment of the rim induced by the hBN moiré pattern was considered. A value of ε_r_ = 4 is assumed for the dielectric constant of h-BN, as proposed by Kim et al. [19] for 2–5 nm thin h-BN films. Using the following formula, the resulting energy shift can be estimated:

[1]
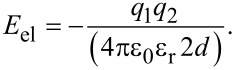


Approximating the ion as a point charge yields an estimate for the energy shift of about 0.64 eV, compared to the measured value of 0.85 eV. One can see that this model gives a good estimate for the energy shift and thus for the screening energy stabilizing the molecular ion on the surface. It should be noted that this can only to be seen as a rough approximation for the energy, because of the uncertainties of the molecular position *d* and the dielectric constant of a single sheet of h-BN.

**Figure 7:**
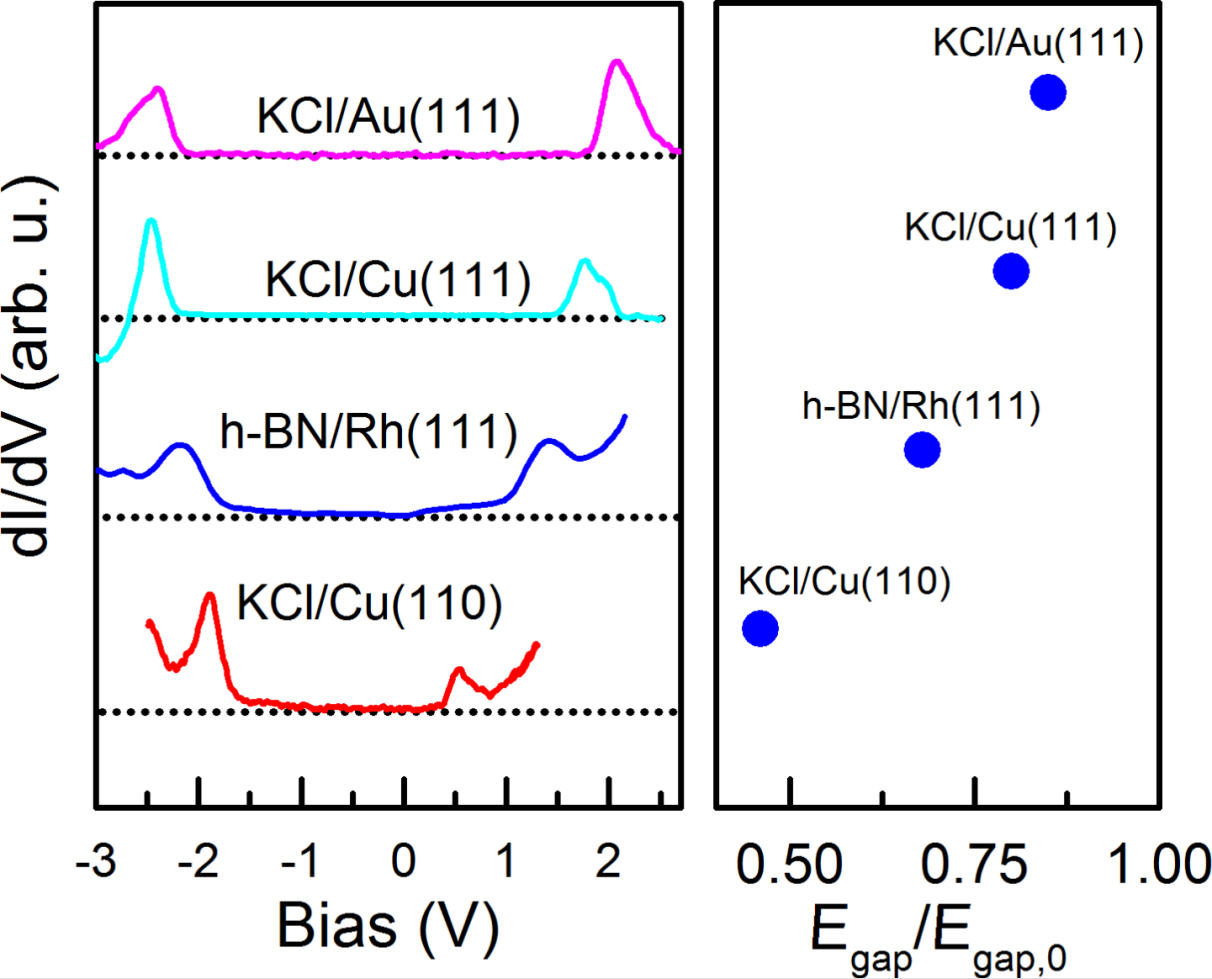
STS measurements of pentacene on various decoupling layers (see legend). In the case of Au(111) and Cu(111), KCl grows as a bilayer, whereas on Cu(110), a monolayer growth was observed. The right graph indicates the deviation of the HOMO–LUMO gap of pentacene on the respective surfaces (*E*_gap_) from its unperturbed gas-phase gap (*E*_gap,0_).

Similar STS measurements of pentacene were carried out on KCl/Au(111), KCl/Cu(111) and KCl/(Cu110). The resulting STS data are depicted in Figure 7.

The HOMO–LUMO gap can be used as a measure of the efficiency of an insulating layer to electronically decouple an adsorbate from the metal substrate. The ratios between the on-surface *E*_i_ − *E*_a_ gaps and the unperturbed gas-phase gaps are listed in Table 1. For a similar decoupling layer (KCl), but various substrates, the decoupling strength decreases with the work function of the substrate.

**Table 1:** Experimental values for *E*_a_, *E*_i_, *E*_gap_/*E*_gap,0_ for pentacene as well as the values for Φ. Φ has been determined experimentally for the KCl layers on the different substrates by *I*(*z*) measurements performed within the Ph.D. Thesis of A. Kabakchiev [20]. For h-BN, a literature value is presented.

pentacene on	*E*_a_ (eV)	*E*_i_ (eV)	*E*_gap_/*E*_gap,0_	Φ (eV)

KCl/Au(111)	1.7	6.15	0.85	3.8
KCl/Cu(111)	0.95	5.14	0.8	2.7
h-BN/Rh(111)	2.75	6.3	0.68	4.15 [16]
KCl/Cu(110)	1.67	4.09	0.46	2.2

**Figure 8:**
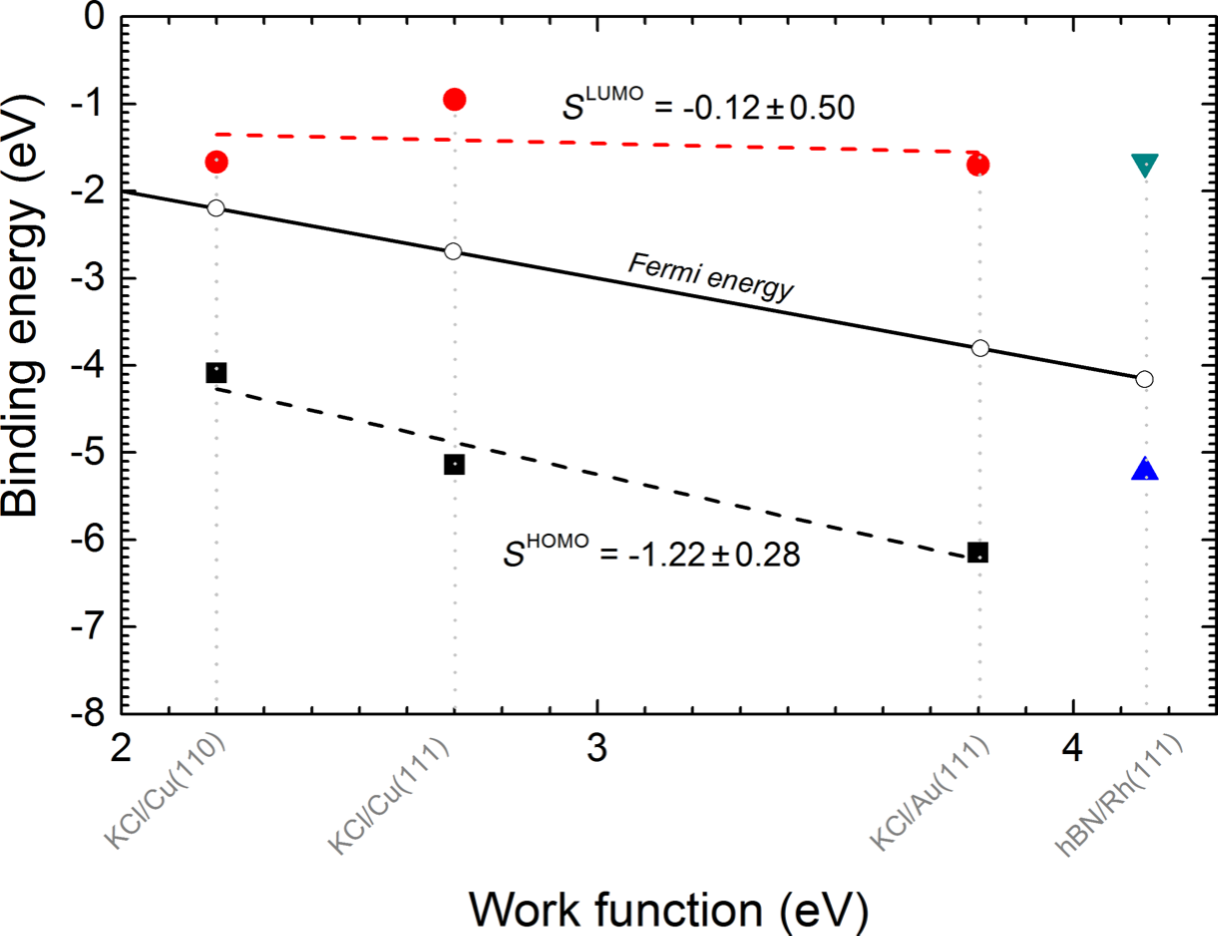
Experimentally determined energies for *E*_a_ (red circles) and *E*_i_ (black squares) of pentacene versus the work function of the respective KCl/metal substrates. Separately indicated are the values for h-BN/Rh(111). Dashed lines indicate linear fits of *E*_a_ and *E*_i_. The open circles indicate the position of the Fermi energy (black line is a linear fit to these values).

Willenbockel et al. stated that larger work functions tend to lead to a larger molecule–substrate spacing upon adsorption [17]. Furthermore the remaining interaction of the pentacene with the respective metal plays an additional role in the decoupling, since the inert Au(111) substrate perturbs the molecular states less than the more reactive Cu(110) substrate. The values for *E*_a_ and *E*_i_ were calculated for all the investigated material systems according to the scheme shown in Figure 6 (Table 1). Plotting *E*_a_ and *E*_i_ versus the work function of the respective substrate yields the graph depicted in Figure 8.

As suggested by Willenbockel et al. [17], HOMO and LUMO do not necessarily interact equally with the substrate. The suggested parameter *S* = d*E*_B_/dΦ, the derivative of the binding energy with respect to the work function, is an indicator for the binding strength. While Willenbockel et al. perform the calculation of *S* both with the clean metal work function, Φ_0_, and with the work function of the molecule covered metal, Φ, our study includes isolated molecules. Therefore the only relevant work function is that of the insulator-covered surface, which we denote by Φ.

Molecular orbitals that interact weakly with the metal will maintain a fixed binding energy with respect to the vacuum level, and thus a value of *S* close to zero. Orbitals that are strongly bound to the metal will be energetically fixed with respect to the Fermi level, and since the Fermi level decreases linearly with the work function, this results in a value of *S* close to −1. A value of *S* < −1 suggests a stabilization mechanism beyond the model, such as bond stabilization due to the shift of binding orbitals towards higher energies [17].

We obtained the values of *S*^HOMO^ = d*E*_i_/dΦ and *S*^LUMO^ = d*E*_a_/dΦ by fitting the data points for *E*_a_ and *E*_i_ of the pentacene/KCl/metal substrates to a linear function. The energy levels of pentacene/h-BN/Rh(111) were not included in the fit due to the different nature of molecule–substrate adsorption in this case, which is discussed below.

For *S*^HOMO^ = −0.12 ± 0.5, a value close to 0 reveals the vacuum-level pinning of *E*_a_, which suggests a weak coupling of this orbital to the substrate. On the other hand, *E*_i_ decreases with increasing work function, with a slope *S*^HOMO^ = −1.22 ± 0.28. In this case, the value of *S*^HOMO^ is close to −1 (within less than a standard deviation of −1), which suggests that the HOMO is Fermi-level pinned and therefore more strongly coupled to the substrate. In summary, this implies that the LUMO is vacuum-level pinned and the HOMO is Fermi-level pinned.

Our results suggest that the approach of Willenbockel et al. for determining the mechanism of orbital pinning for HOMO and LUMO [17] is not limited to molecules adsorbed onto bare metals, but is also valid for single molecules on metals covered with a thin decoupling layer having homogeneous properties. An exception is found for the HOMO on h-BN where the intrinsic dipole moment of the molecule within the depressions in the h-BN superstructure modifies the coupling strength between molecule and substrate.

Our observations show how the energies of the molecular orbitals of pentacene adsorbed on h-BN/Rh(111) deviate from the trend set by pentacene on KCl/metal (Figure 8). This deviation reflects the unique characteristics of the adsorption of molecules on h-BN/Rh(111), namely the corrugated surface and attraction by local electric fields parallel to the surface. Lateral electric fields have a stabilizing effect on the molecular orbitals, which, for a symmetrical orbital, depend on its polarizability [21]. In addition, the molecules appear curved as a result of both, the h-BN curvature and the curved geometry of the attractive electric field. These unique properties of h-BN/Rh(111) can explain why the HOMO of pentacene deviates from the trend set by pentacene on KCl/metal.

## Conclusion

In conclusion, we confirm for the system of pentacene on h-BN/Rh(111) the effective decoupling properties of h-BN. The h-BN layer offers a desirable capacity to immobilize molecules inside the valleys on the surface, even at room temperature. The trapping capacity stems from an interaction with in-plane electric fields present on the substrate. This enhances the molecule–substrate interaction, which decreases the HOMO–LUMO gap in comparison to the same molecule adsorbed on KCl/metal surfaces. In spite of this interaction, h-BN/Rh(111) provides sufficient electronic decoupling to allow for the observation of clear pentacene HOMO and LUMO peaks by STS. Furthermore, STS mapping at the peak energies reveals the shapes of the corresponding molecular orbitals, and tip functionalization further improves the contrast of these structures.

In order to further rationalize the results obtained by STS on pentacene on the h-BN/Rh(111) substrate, comparative studies using a different decoupling layer, namely KCl on varying metal substrates, were performed. For pentacene on KCl/metals, it is surprising that in spite of the complexities of molecule–metal adsorption, the work function of the surface plays a decisive role in determining the energies of the binding molecular orbitals. The observed trends indicate that HOMO and LUMO participate to different extents in binding to the surface. The binding energy of the HOMO is unchanged with respect to the Fermi energy on all three KCl surfaces, indicating an enhanced degree of interaction with the metallic substrate. The LUMO, on the other hand, keeps a fixed position with respect to the vacuum level, suggesting a weaker interaction with the metal.

The results reported here illustrate the benefits of comparing spectroscopic data from the same molecule on similar substrates. Thus, a trend can be observed in the relation between orbital energies and work function, which reveals the physical properties of the molecule and the substrates that are relevant to adsorption. In addition, a deviation from this trend for hBN/Rh(111) helps rationalize its enhanced trapping capacity and its uncommon interaction mechanism.

Our study might be of special interest for the field of molecular electronics, where a precise knowledge of the energetic positions of the available states is of great importance.

## Experimental

All samples were prepared under ultra-high-vacuum (UHV) conditions at a base pressure of 3·10^−10^ mbar and investigated in a custom-built STM operating at a temperature of 5.3 K in UHV.

A Rh(111) single crystal was cleaned by an initial Ar^+^ bombardment at 1103 K for 1 h and a subsequent series of repetitive Ar-ion bombardments (15–30 min) at room temperature, followed by annealing at 1073 K for 5 min.

A h-BN layer was grown by chemical vapor deposition (CVD) by heating the Rh(111) sample to 1073 K and exposing it to 110 L of borazine ((HBNH)_3_) gas. h-BN grows in a self-terminating growth process [22] on the (111) surface of the crystal. The KCl layers on various metal surfaces were generated by thermal evaporation of KCl at 653 K for 20 min. During this process, the substrates were kept at room temperature. The metal substrates (Au(111), Cu(111), Cu(110)) were cleaned by alternating sequences of Ar-ion bombardment and annealing at 843 K. The annealing temperature was reduced by 50 K for the last cycle.

Pentacene molecules were deposited by thermal sublimation at 458 K. The deposition time was adjusted to achieve submonolayer coverage with individual pentacene molecules on the sample surface. During the pentacene deposition, the substrate was kept at room temperature.

During STM, all bias voltages were applied with respect to the sample, meaning that for negative bias voltages, electrons tunnel from the sample to the tip. For STS measurements, the differential conductance was recorded utilizing a lock-in amplifier. The bias voltage was modulated with an amplitude of 50 mV at a frequency of 832 Hz.
